# Increasing the angle between caudal screw and the transverse plane may aggravate the risk of femoral head necrosis by deteriorating the fixation stability in patients with femoral neck fracture

**DOI:** 10.1186/s40001-024-01737-3

**Published:** 2024-03-12

**Authors:** Jian Zhang, Shengyu Wan, Xiaozhong Luo, Caidong Zhang, Chao Wu, Lipeng He, Jingchi Li

**Affiliations:** 1 Department of Orthopaedics, Zigong Fourth People’s Hospital, Zigong, 643000 Sichuan People’s Republic of China; 2 Department of Orthopedics, Luzhou Key Laboratory of Orthopedic Disorders, The Affiliated Traditional Chinese Medicine Hospital, Southwest Medical University, NO.182, Chunhui Road, Longmatan District, Luzhou, 646000 Sichuan People’s Republic of China; 3 Department of Orthopaedics, Wuxi Hospital of Traditional Chinese Medicine, Wuxi, 214000 Jiangsu People’s Republic of China

**Keywords:** Femoral neck fracture, Femoral head necrosis, Finite element analysis, Screw trajectory optimization, Fixation stability

## Abstract

Necrosis of the femoral head is the main complication in femoral neck fracture patients with triangle cannulated screw fixation. Instant postoperative fixation instability is a main reason for the higher risk of femoral head necrosis. Biomechanical studies have shown that cross screw fixation can effectively optimize fixation stability in patients with proximal humerus fractures and pedicle screw fixation, but whether this method can also effectively optimize the fixation stability of femoral neck fractures and reduce the corresponding risk of femoral head necrosis has yet to be identified. In this study, a retrospective review of imaging data in femoral neck fracture patients was performed. The cross angle between the femoral neck and the caudal cannulated screw was reported; if the angle between the screw and the transverse plane increased, it was recorded as positive; otherwise, it was recorded as negative. Angle values and their corresponding absolute values were compared in patients with and without femoral head necrosis. Regression analysis identified potential risk factors for femoral head necrosis. Moreover, the biomechanical effect of the screw–femoral neck angle on fixation stability was also verified by numerical mechanical simulations. Clinical review presented significantly larger positive angle values in patients with femoral head necrosis, which was also proven to be an independent risk factor for this complication. Moreover, fixation stability progressively deteriorated with increasing angle between the caudal screw and the transverse plane. Therefore, increasing the angle between the caudal screw and the transverse plane may aggravate the risk of femoral head necrosis by deteriorating the fixation stability in patients with femoral neck fracture.

## Introduction

Femoral neck fracture is a common type of injury, and internal fixation operations can effectively treat this disease in young and middle-aged patients [1, 2]. However, femoral head necrosis is a main complication in femoral neck fracture patients after internal fixation operations [3, 4]. Recently, surgeons have paid more attention to the effect of fixation stability on the risk of femoral head necrosis [5, 6]. Specifically, poor fixation stability causes micromotion on fracture interfaces, hindering the healing of fractures and the restoration of blood supply to the femoral head [7, 8]. Therefore, risk factors related to poor fixation stability (i.e., larger Pauwels angle) were reported to be risk factors for femoral head necrosis [4, 6]. Correspondingly, surgical techniques that can better restore fixation stability may be effective methods to reduce the risk of femoral head necrosis.

Triangle cannulated screw fixation is the most commonly used surgical technique when treating femoral neck fractures [9, 10]. Traditionally, the trajectory of cannulated screws was coaxial to the long axis of the femoral neck [11, 12]. The biomechanical significance of screw configuration strategies has been reported repeatedly. Changes in the screw insertion angle were an important factor affecting fixation stability [11, 12]. Moreover, biomechanical studies on proximal humeral fracture and vertebral burst fracture fixations proved that the cross screw configuration strategy could effectively optimize instant postoperative angular stability [13, 14]. The biomechanical significance of cross-angle cannulated screws has also been investigated. The computational results recorded an alleviated stress concentration tendency in the model with cross-angle fixation. Whether this screw configuration strategy can optimize the instant postoperative stability and reduce the corresponding risk of femoral head necrosis and the effect threshold of the cross angle have yet to be identified. Identifying these topics could provide theoretical foundations for the optimization of triangle cannulated screw fixation and the corresponding prognosis of femoral neck fracture patients.

## Materials and methods

### Clinical review

#### Patient collection

**Fig. 1:**
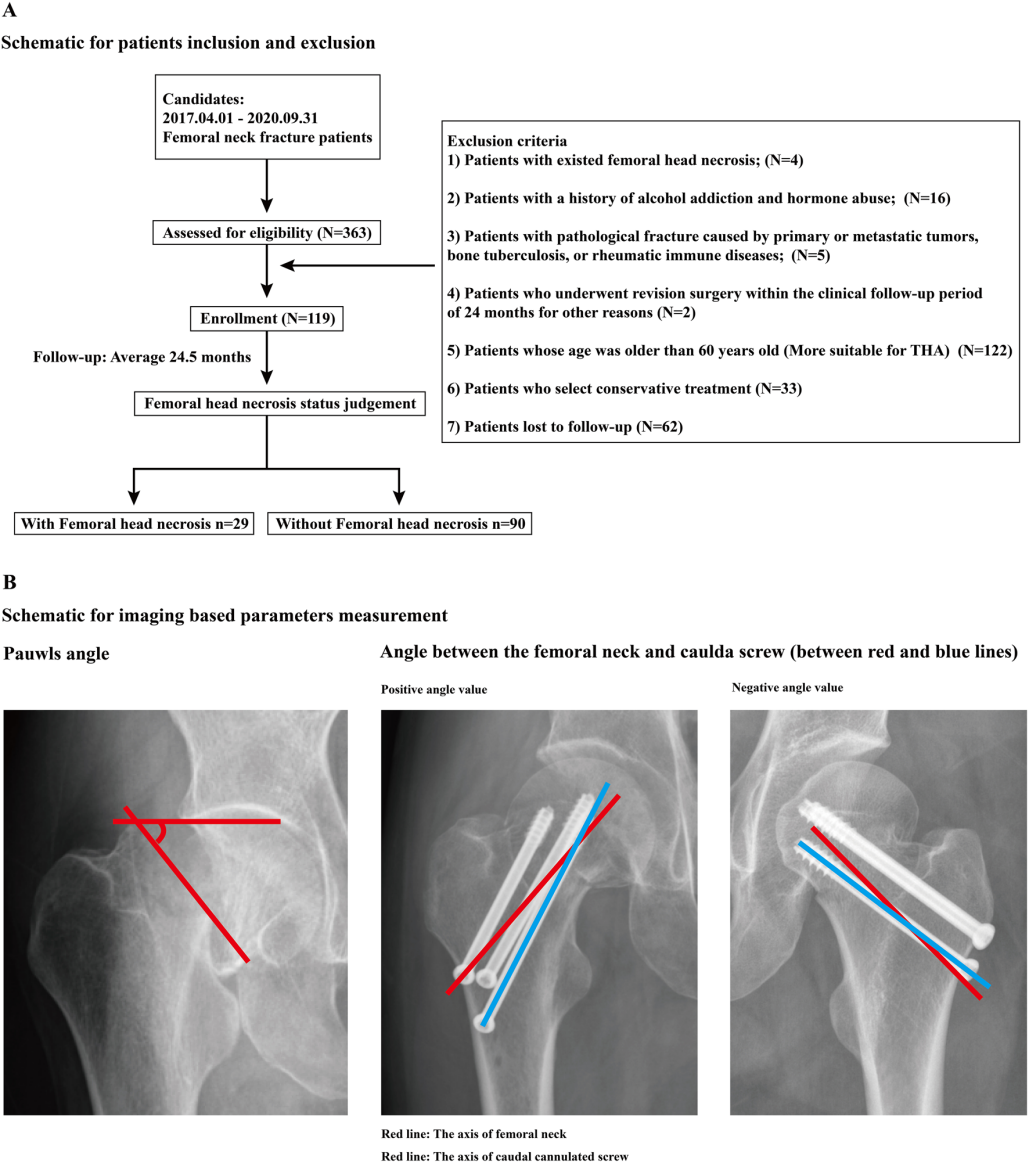
Schematic for patient inclusion and exclusion, and the measurement of imaging-based parameters

**Fig. 2:**
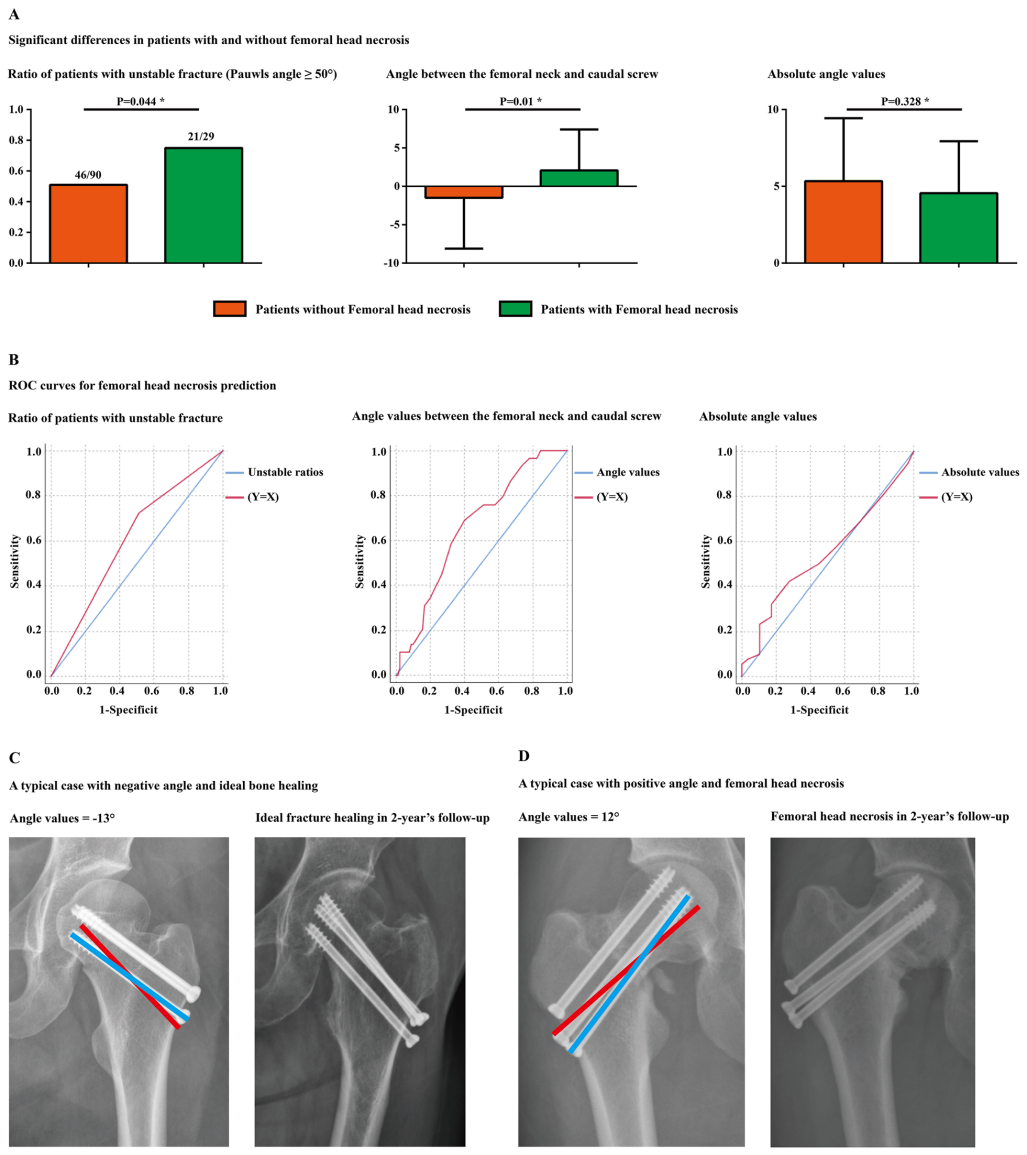
Significant differences in the indicators between the patient groups with and without femoral head necrosis, ROC curves for femoral head necrosis predictions, and typical cases with different caudal screw–femoral neck angles and different clinical prognoses (with and without necrosis of the femoral head)

The ethics committees of our hospital reviewed and approved the protocol of this study (2023-009). We retrospectively reviewed patients who suffered femoral neck fracture and underwent triangle cannulated screw fixation from April 2017 to September 2020. The inverse triangle cannulate screw fixation was the only used internal fixation operation in the patient inclusion period. Therefore, only patients with this operation strategy was enrolled in this study. The exclusion criteria were as follows: (1) patients with existing femoral head necrosis; (2) patients with a history of alcohol addiction and hormone abuse; (3) patients with primary or metastatic bone tumors, bone tuberculosis, or rheumatic immune diseases; (4) patients who underwent revision surgery within the clinical follow-up period of 24 months for other complications (i.e., screw pull-out or breakage); and (5) patients whose age was older than 60 years old (these patients are more suitable for total hip arthoplasty than triangle cannulated screw fixation). The age, sex, and BMI of these patients were recorded. A well-trained orthopedic surgeon performed all operations. Screw types and sizes were identical in these patients. The tip–apex distance of all enrolled patients was smaller than 25 mm (Figs. 1, 2) [4, 15].

#### Radiographic data collection

All patients underwent anterior–posterior radiography for the last two times, including immediately postoperatively and 2 years after the screw fixation operation [16, 17]. The angle of the caudal screw and the femoral neck were measured in the instant postoperative imaging data. If the angle between the screw and the transverse plane increased, it was recorded as positive; otherwise, it was recorded as negative. Angle values and their corresponding absolute values were recorded separately. Moreover, the Pauwels angle of these patients was measured in the preoperative imaging data. Fractures with Pauwels angles larger than 50° were defined as instability fractures and vice versa. Patients who suffered femoral head collapse or subcortical cysts in the 2-year follow-up imaging data were diagnosed with necrosis of the femoral head [4, 18].

#### Statistical analyses

Radiographic and demographic indicators are presented as the mean ± standard deviation for continuous variables and number (percentage) for categorical variables. We conducted statistical analyses in SPSS software. The intraclass correlation efficiency (ICC) was computed to identify the repeatability of continuous variables [19], including the Pauwels angle and the cross angle between the femoral neck and caudal screw. An ICC ≥ 0.8 represents excellent reliability [20, 21].

When comparing the difference between groups with and without femoral head necrosis, the independent samples Student’s *t* test was used for continuous variables, and the Chi-square test was used for the categorical variables [22, 23]. We performed binary logistic regression to identify independent risk factors for femoral head necrosis. Univariate analyses of each potential risk factor were performed, and the variables that achieved a significance level of *P* < 0.1 were entered into multivariate analyses. Variables with *P* < 0.05 were considered independent risk factors in the multivariate analysis [24, 25]. Finally, we performed ROC curve analyses to assess the predictive value of potential risk factors, and the area under the curve (AUC) was calculated as an indicator to judge the predictive performances of independent risk factors [25, 26]. A *P* value less than 0.05 indicated a significant difference.

### Numerical surgical simulations and finite element analysis (FEA)

#### Construction of the intact finite element (FE) model

The proximal femur model was constructed based on the outline of the syn-bone model rather than that of any special patient. The model construction strategy was selected to avoid ethical procedures and eliminate the confounding effect caused by individual differences in outlines in different patients [27, 28]. A thin slice thickness CT scan was performed in the syn-bone femur model (thickness = 0.55 mm). The range of the proximal femur was defined from the tip of the femoral head to 30 cm below the lesser trochanter [28, 29]. The outline of the proximal femur model was constructed according to the CT-scanned femur outline in 3D-CAD software. Consistent with our published studies. The computational efficiency and accuracy of the numerical model constructed by this method were better than those of the traditional reverse model construction strategy [30, 31]. Cortical and cancellous bone were separately constructed, and outlines of these bony structures were separately constructed based on the CT imaging data [27, 28].

#### Construction of triangle screw fixed femoral neck fracture models with different caudal screw–femoral neck angles

To simulate the fixation stability of different screw fixation strategies in the instability fracture model, the Pauwels III type femoral neck fracture was used in the proximal femur model. The Pauwels angle of fracture was set as 55°. The fracture was located in the middle position of the femoral neck, and a 1-mm crevice was constructed to simulate the fracture line. The numerical model of a semi-thread lag cannulated screw was also constructed in 3D-CAD software. When simulating the inverse triangle screw fixation operation, two cranial cannulated screws were inserted along the trajectory coaxial to the long axis of the femoral neck. When inserting the caudal side cannulated screw, the screw trajectory was parallel to the femoral neck in both transverse and sagittal planes.

**Fig. 3:**
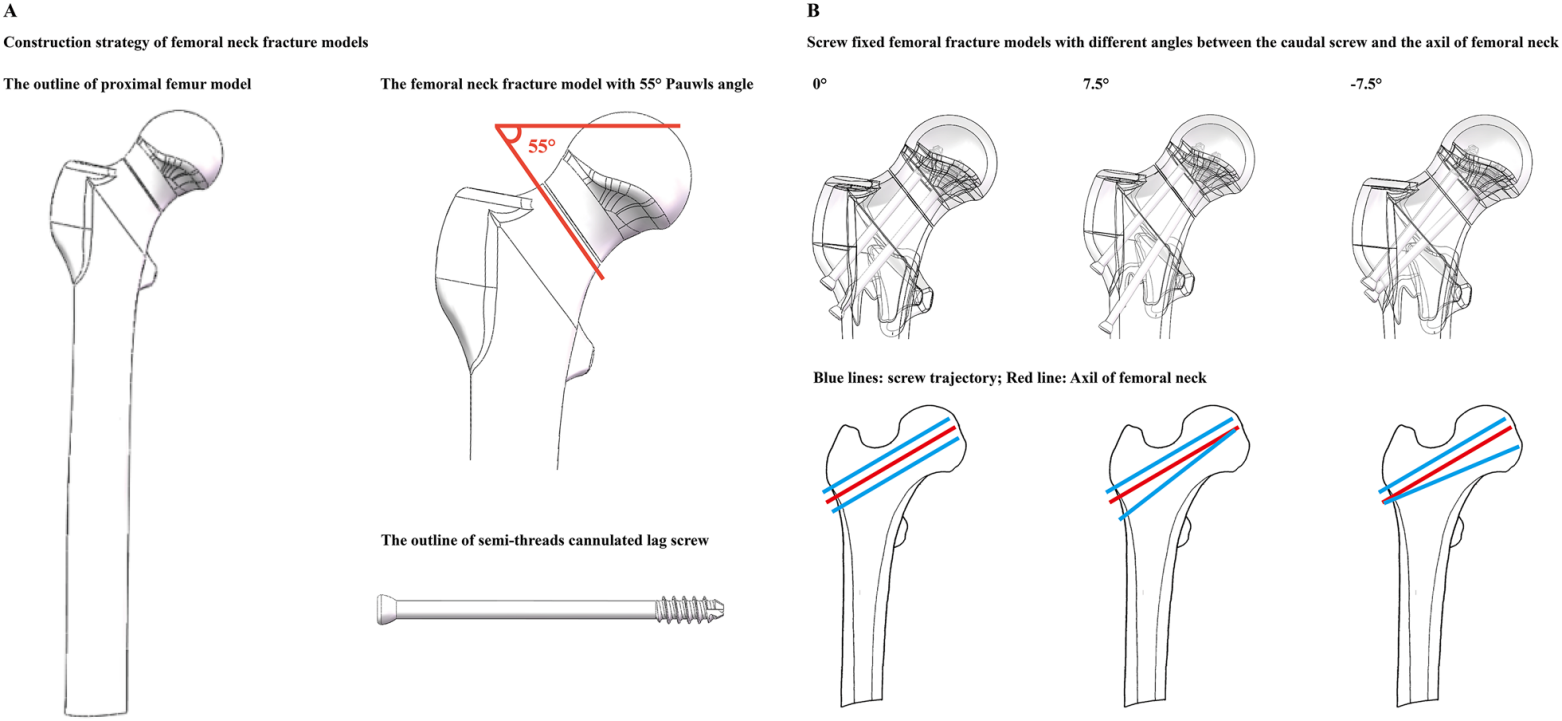
Schematic for numerical model construction, and surgical simulations in models with different caudal screw angles

To simulate different grades of cross fixation, the angle of the caudal screw and femoral neck on the coronal plane was adjusted. Five screw-fixed femoral neck fracture models were constructed. The cross-angle adjustment ranged from 0° to ± 7.5°. Detailed screw cross angles in different models are presented in Fig. 3.

#### Boundary and loading conditions

**Fig. 4:**
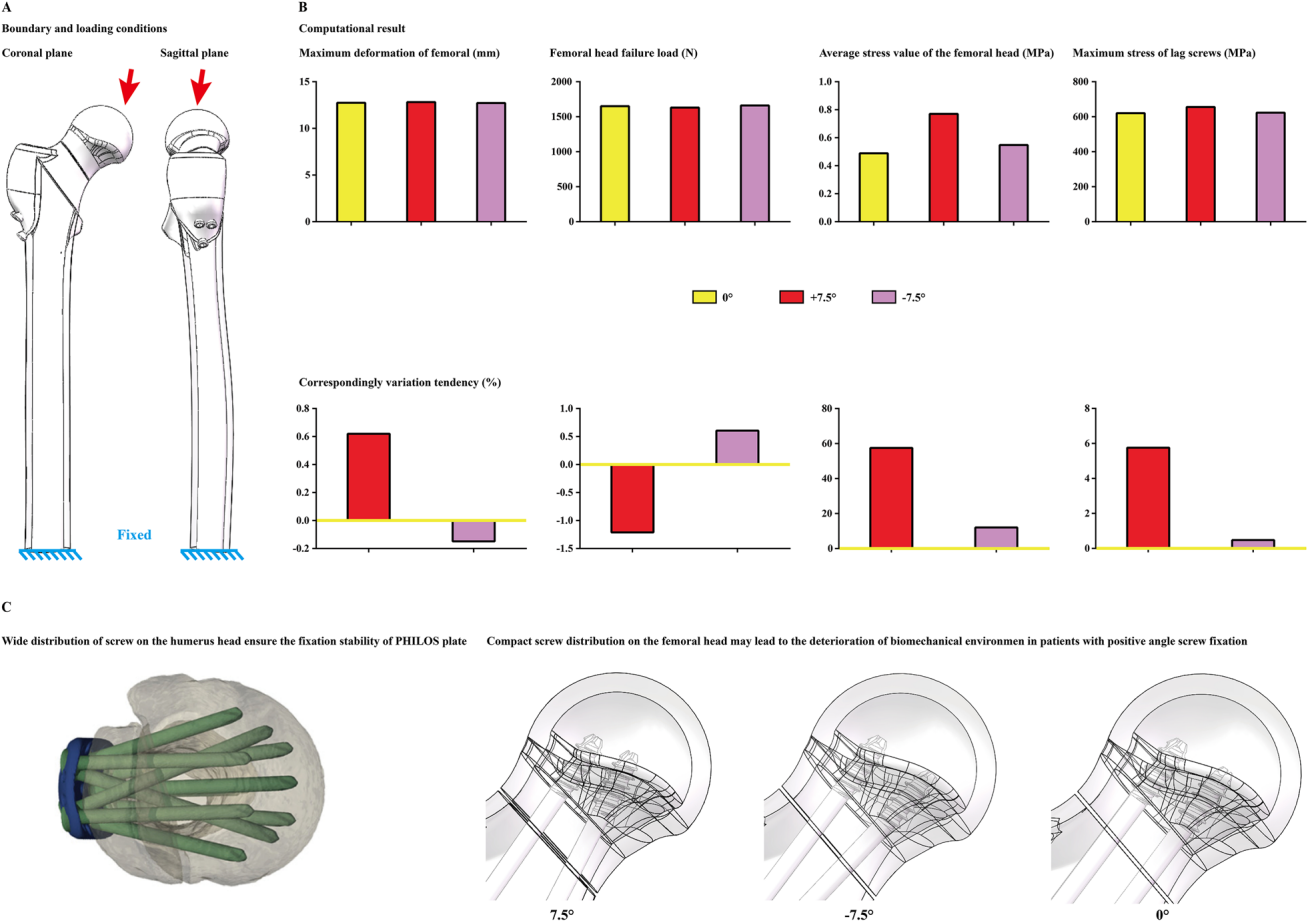
Boundary and loading conditions for numerical simulation, computational results in different models, and schematic for the hypothesis that the compact screw distribution may lead to the deterioration of biomechanical environment in patients with positive angle screw fixation

Material properties of cortical and cancellous bone and titanium alloy cannulated screws were separately defined as isotropic material according to the same type studies [32, 33]. The degrees of freedom of the inferior surfaces of the numerical models were completely fixed [34, 35]. Different forces were applied to the femoral head at 10° laterally on the coronal plane and 9° posteriorly on the sagittal plane [27, 28]. The applied load was continuously increased under a 50 N step until 2100 N, and the maximum femoral head deformation and stress distribution on the screw and femoral head were recorded under a 2100 N compressive load. The compressive load when the maximum deformation of the femoral head reached 10 mm was also recorded in this study. The corresponding force value was recorded as the failure strength of screw fixation (Fig. 4) [27, 29].

## Results

### Retrospective review of clinical data

#### Patient collection and significant difference verification

A total of 119 femoral neck fractures treated by inverse triangle cannulated screw fixation were enrolled in this study. The overall incidence rate of femoral head necrosis was 24.37%. The interobserver and intraobserver reliability of the continuous variables were excellent, with ICCs of 0.887 and 0.842, respectively. There were no significant differences in demographic covariates (age, sex, and BMI) between patients with and without femoral head necrosis. Meanwhile, the difference in the absolute value of angles was insignificant between the two groups. In contrast, patients with femoral head necrosis in the 2 year follow-up suffered significantly larger positive angle values and a significantly higher incidence rate of instability fracture type (Pauwels angle ≥ 50°) (Table 1 and Fig. 2).

**Table 1:** Significant comparison between variates for patients with and without femoral head necrosis

	Without femoral head necrosis	With femoral head necrosis	*P* value
Age	50.53 ± 9.39	51.28 ± 13.14	0.739
Sex (male/female)	40/50	11/18	0.538
BMI	21.51 ± 2.75	21.69 ± 3.06	0.765
stability status (Pauwels angle < or ≥ 50°)	44/46	8/21	0.044*
Cross angle	− 1.49 ± 6.62	2.07 ± 5.34	0.01*
Absolute value of cross angle	5.38 ± 4.1	4.55 ± 3.39	0.328

^*^Statistical significance (*P* < 0.05)

#### Independent risk factors and parameter prediction values for femoral head necrosis

When identifying independent risk factors for femoral head necrosis, based on the results of univariate logistic regression analyses, a higher instability fracture rate and larger angle values (rather than larger absolute angle values) were entered into the multivariate analysis. The results showed that only a larger positive angle was an independent risk factor for femoral head necrosis. We performed ROC curve analyses to assess the predictive value of a higher rate of instability fracture, larger angle values and absolute angle values while predicting femoral head necrosis risk. The AUCs of these three parameters were 0.607, 0.661, and 0.545, respectively (Tables 2, 3, and Fig. 2).

**Table 2:** Logistic regression analysis of AVF

	OR	95% CI		*P* value
Univariate analyses				
Age	0.993	0.952	1.035	0.737
Sex (male/female)	0.764	0.324	1.801	0.538
BMI	0.977	0.842	1.134	0.763
Stability status (Pauwels angle < or ≥ 50°)	0.398	0.16	0.993	0.048^#^
Cross angle	0.913	0.851	0.98	0.012^#^
Absolute value of cross angle	1.058	0.945	1.185	0.327
Multivariate analysis				
Stability status (Pauwels angle < or ≥ 50°)	0.471	0.184	1.204	0.116
Cross angle	0.921	0.857	0.991	0.027*

^#^Variables that achieved a significance level of *P* < 0.1 in the univariate analysis

^*^Statistical significance (*P* < 0.05)

**Table 3:** The cut-off value, sensitivity and specificity for femoral head necrosis prediction

	Stability status	Cross angle	Absolute value of cross angle
Cut-off value	1.5	− 0.5	6.5
Sensitivity	0.724	0.724	0.422
Specificity	0.489	0.544	0.724
AUC	0.607	0.661	0.545

#### Numerical mechanical simulations

The maximum displacement values and stress distribution of the femoral head and lag screws were computed and recorded under a 2100 N compressive load, and the failure loads of different models were also computed in this study. The results show that there were slight differences in the computed values in models with 0° and − 7.5° screw fixation angles. However, compared to these two models, poor fixation stability and stress concentration can be observed in the model with 7.5° screw fixation. The femoral head stress value in the 7.5° screw-fixed model increased by more than 60% compared to that in the 0° screw-fixed model. Moreover, the failure loads of all computed models were less than 2100 N in this study (Figs. 3, 4).

## Discussion

To investigate whether changes in the caudal screw insertion angle affect the instant postoperative fixation stability and corresponding risk of femoral head necrosis in femoral neck fracture patients with inverse triangle screw fixation, comprehensive research consisting of a clinical review and corresponding biomechanical numerical simulations was performed. A significantly larger positive angle (larger angle between the caudal screw and transverse plane) of the caudal screw was proven to be an independent risk factor for femoral head necrosis, and a larger femoral deformation value and instant postoperative biomechanical environment deterioration were also recorded in models with a positive angle of the caudal screw. Considering that poor instant postoperative fixation stability (which can be well reflected by computed displacement and stress values) is an important risk factor for femoral head necrosis, increasing the angle between the caudal screw and the transverse plane should be avoided when designing the trajectory of the caudal screw to the transverse plane to reduce the risk of femoral head necrosis biomechanically.

In this study, a higher absolute value of the cross angle between the femoral neck and the caudal screw could not effectively optimize the instant postoperative fixation stability and was not an independent predictor for a lower risk of femoral head necrosis. Theoretically, angular stability can be effectively optimized in models with cross-angle fixation, and this topic has been repeatedly verified in fracture models [36, 37]. Therefore, we suspected that larger absolute screw angle values (either positive or negative) could improve fixation stability. However, inconsistent with our anticipation, larger positive angle fixation of the caudal screw did not optimize the fixation stability; in contrast, it increased the risk of femoral head necrosis biomechanically.

In this study, positive screw angle triggers poor fixation stability and a higher risk of femoral head necrosis, this may root in the limit screw distribution in the femoral head. Specifically, compared to the traditional locking plate, proximal humerus models fixed by the PHILOS plate present better stability [13, 14]. Biomechanical research present that the abduction angulation of screw insertion cause a wider screw distribution in the humerus head, which may be an important reason for better angular stability [38, 39]. Correspondingly, in femoral neck fracture models with negative angle caudal screw fixation, there is also an abduction angle between cranial and caudal screws, resulting in a wider distribution of screws in the femoral head may also provide better angular stability. In contrast, in patients with positive angle screw fixation, adduction between cranial and caudal screws triggers limited screw distribution. This maybe an important reason for poor fixation stability. In other words, screw distribution in the femoral head, rather than the absolute value of screw cross angles, may be an important factor for fixation stability. Besides, difference in biomechanical environment existed in fracture with screw and locking plate fixation. Therefore, another contribution of this study was we prove that angular screw insertion can also optimize the fixation stability in patients with screw fixation, and which can be promoted in the treatment of other fracture type with screw fixation.

Additionally, 2100N is the normal load on the femur of a 70 kg person walking on one leg (3 times body weight), but the failure load of all models were less than this value in the current study. Given that the stability of screw fixed fracture stepwise optimized within the bone healing process, based on the current computational result, we believe that regardless of the fixation angle used, instant-postoperative weight bearing should was not recommended in femoral neck fracture patients to avoid fixation failure, and corresponding risk of femoral head necrosis [40, 41]. And this topic also should been verified in our subsequent studies.

Admittedly, following limitations should be clarified. Firstly, although the incidence rate of unstable fracture was significantly higher in patients with femoral head necrosis, this factor was not an independent risk factor for femoral head necrosis. This critical positive result may root in the limited sample size. And which will not deny the adverse effect of unstable fracture type on the risk of femoral head necrosis. Besides, the outline of femoral numerical model was defined according to the syn-bone model, rather than any patients. This model construction strategy can effectively eliminate the confounding effect of individual difference in femoral structure outline on computational results. But from another perspective, the biomechanical significance of these factors can also not been identified in this study.

Meanwhile, it was a commonsense that the fracture angle (Pauwels angle) can significantly affect the postoperative stability in femoral neck patients with triangle screw fixation, and this topic has also been validated in the current study. Besides, during the model construction strategy, only 55° of Pauwels angle was selected to construct the femoral neck fracture model. That’s because this degree is commonly observed in patients with Pauwels III type of fracture (instability fracture) [4, 6]. This model construction strategy ignore the effect of Pauwels angle change on fixation stability, and which was an existed limitation of this study. However, the main topic of this study was to investigate changes in screw insertion angle on fixation stability and corresponding risk of femoral head necrosis. Constructing the instability fracture model can to avoid the effect of screw angle changes on stability being overshadowed by the stability of the fracture itself. This model construction strategy (choose fracture type that are more likely to lead to complication) have been widely used in the same type studies [27, 29]. Therefore, we believe this model construction protocol can meet the demand of numerical mechanical simulation’s necessity of the current study.

Moreover, based on this study, a larger negative angle (more parallel to the transverse plane) of the caudal screw could effectively optimize the fixation stability and reduce the risk of femoral head necrosis, but if this conclusion also suitable for cranial two screws, and whether the increase in negative angles optimize the fixation stability within a specific threshold range have yet to be identified in this study. These topics should be verified by our future clinical and biomechanical researches with larger sample sizes and more complex model construction strategies.

## Conclusions

By comprehensively performing clinical and biomechanical research, this study proved that increase the angle between caudal screw and the transverse plane can aggravate the risk of femoral head necrosis by deteriorating the fixation stability in patients with femoral neck fracture. Therefore, the trajectory of caudal screw to the transverse plane to reduce the risk of femoral head necrosis biomechanically.

## Data Availability

All the data of the manuscript are presented in the paper.
